# Developing Digital Platforms to Help Older Adults Stay Physically and Socially Active: Co-Design Study to Develop a Toolkit and Evaluate Its Impact

**DOI:** 10.2196/89785

**Published:** 2026-08-27

**Authors:** Leonie Cooper, Hannah Bradwell, Colin B Shore, Simone A Tomaz, Rory Baxter, Gemma C Ryde, Swen E Gaudl, Richard Haynes, Catherine Hennessy, Ray B Jones, Anna C Whittaker, The GOALD Project, Catherine H. Hennessy

**Affiliations:** 1 Faculty of Health, University of Plymouth, Plymouth, England, United Kingdom; 2 School of Health and Life Sciences, Glasgow Caledonian University, Glasgow, United Kingdom; 3 Faculty of Health Sciences and Sport, University of Stirling, Stirling, Scotland, FK4 9LA, United Kingdom, 44 01786467816, 44 01786467816; 4 School of Cardiovascular and Metabolic Health, University of Glasgow, Glasgow, Scotland, United Kingdom; 5 Department of Applied IT, University of Gothenburg, Gothenburg, Västra Götaland, Sweden; 6 Faculty of Arts and Humanities, University of Stirling, Scotland, United Kingdom; 7 Faculty of Social Sciences, University of Stirling, Scotland, United Kingdom; 8 See Acknowlegments

**Keywords:** aging, co-design, digital health, older adults, physical activity, toolkits, user-centered design, virtual reality

## Abstract

**Background:**

Digital technologies have the potential to support physical, cognitive, and social activity among older adults, but many small- and medium-sized enterprises (SMEs) lack the resources to conduct meaningful co-design with older adults. Toolkits derived from rigorous co-design processes may offer a scalable mechanism for translating end-user priorities into real-world product development.

**Objective:**

The Generating Older Active Lives Digitally study aimed primarily to engage older adults in a co-design process to identify priorities for digital technologies that support physical activity and reminiscence. Secondary objectives were to (1) translate these findings into a practical developer-facing toolkit, and (2) evaluate the toolkit’s perceived utility and influence among digital technology SMEs.

**Methods:**

A total of 157 participants (121 older, 7 younger, and 29 staff) across 15 community and care settings in England and Scotland engaged in 106 technology interaction sessions, 22 evaluation focus groups, and 10 co-design workshops. These involved more than 20 digital technologies. Thematic analysis and structured card-ranking tasks were used to derive their priorities. Preliminary toolkits were co-designed and used by 10 UK-based SMEs who received small grants to apply the toolkit to active development projects. Their reports and follow-up interviews were analyzed thematically to identify perceived impacts on design decisions, product adaptations, and business outcomes.

**Results:**

Co-design activities generated 7 cross-cutting themes: motivation, content, barriers, design and inclusivity, suitability, acceptability, and motivations to use. These were organized into 3 toolkit sections: general design principles, online physical-activity platforms, and virtual reality. SMEs reported that the toolkits enhanced understanding of older adults’ needs, validated design decisions, and inspired some new features. Perceived impacts included improved usability, expanded accessibility options, increased content variety, clearer instructional design, enhanced social components, and reduced operational costs. SMEs also reported some business benefits, including strengthened cases for investment and increased product uptake.

**Conclusions:**

Co-designed toolkits may offer a useful and cost-effective mechanism for translating older adults’ priorities into digital product development. SMEs perceived this toolkit as practical, relevant, and impactful for informing design choices. This approach may complement, but does not replace, direct user involvement. It may help accelerate inclusive digital-health innovation for aging populations.

## Introduction

### Background

It is expected that by 2030, a total of 1 in 6 people in the world will be aged older than 60 years, and by 2050 the world’s older population will have doubled [1]. This demographic shift is placing increasing pressure on health and social care systems, leading to a growing emphasis on promoting healthy aging across the life course [2]. A key focus within this agenda is promoting physical activity within older populations given the role it can play in maintaining and improving physical function [3]. Another area of focus is that of building older adults’ social connectedness and well-being through reminiscence, which has been shown to benefit well-being [4]. Sports-based reminiscence has been particularly successful in men with cognitive impairment, providing, among other mechanisms, a source of specific interest on which to hinge opportunities to increase social participation [5].

Digital technologies have been proposed as a means of supporting physical, cognitive, and social activity in later life [6]. National policies, such as the UK government Consensus on Healthy Aging [7], promote physical, cognitive, and social activity among older adults through exercise, games, reminiscence, and social connection, with digital tools playing a key role. In response, a wide range of digital technologies has emerged, including online exercise platforms, mobile apps, exergames, and immersive virtual reality (VR) experiences. Recent reviews [8,9] covered 16‐22 studies on technologies to support physical activity, while another study [10] identified 13 apps and 24 websites for promoting strength and balance exercises. Positive effects of online exercise platforms or programs designed for older adults have been found for body strength, flexibility, and balance [11,12]. More recently, VR tools designed to support physical activity and reminiscence in older adults have shown promising benefits. These include improved physical rehabilitation at home [13], positive influences on cognition, memory, and depression [14], and enhanced cognitive and social engagement through reminiscence experiences [11,15]. Despite these advances, older adults often experience challenges when adopting and sustaining engagement with digital technologies. Many report positive attitudes toward the potential usefulness of technology to increase their physical and social activity [16]. However, barriers related to usability, accessibility, confidence, cost, security, and perceived relevance frequently result in poor adoption or disengagement [17]. Evidence suggests that when technologies are well-designed, have clear training or guidance, have adaptations specifically for older adults, highlight the benefits, and focus on an older person’s values, conditions, and reducing age-related risks, they can successfully adopt and make use of them [16,18]. However, research has identified the disconnect between what a technology developer may believe an older adult would like or need from the technology and what older adults prioritize [19]. They need to understand the needs and preferences of older adults in using digital products [20-22].

Co-design is widely regarded as a gold-standard approach for addressing this disconnect in digital development. It is the practice of people collaborating and connecting their knowledge, skills, and resources to carry out a design task and to produce new knowledge as people experiment with new ideas [23]. It differs fundamentally from user surveys and market research in that it positions users as active partners in the design process rather than passive respondents. Surveys and market research are valuable for identifying preferences, patterns, and trends, but are typically developer-led and often constrained by predefined questions. In contrast, co-design engages users in generative activities such as ideation, critique, and prototyping. This enables the incorporation of lived experience and tacit knowledge into intervention development. This participatory approach has been associated with improved relevance, acceptability, and implementation of health interventions (eg, other studies [24,25]). As a result, co-design supports successful and relevant design [26] and business growth [27]. However, meaningful co-design can be resource-intensive, requiring time, expertise, and sustained engagement that may be beyond the capacity of small and medium-sized enterprises (SMEs). Further, evidence suggests that poorly implemented co-design offers little advantage over standard design practices [28]. Consequently, alternative mechanisms are needed to support SMEs in integrating end-user insights without requiring them to conduct extensive co-design activities independently.

Despite growing evidence that digital technologies can support physical activity, reminiscence, and social connection among older adults, there remains a gap between older adults’ lived experiences and the design priorities of digital technology developers, particularly SMEs. While participatory co-design can generate rich, contextual insights, resource constraints limit SMEs’ ability to engage in sustained co-design processes. Consequently, there is a need for mechanisms that can translate rigorous co-design insights into forms that are accessible, practical, and usable within real-world development contexts, alongside empirical evaluation of whether such mechanisms meaningfully support development practice. This study seeks to address this gap.

One mechanism is the use of toolkits. In this context, a toolkit refers to a structured collection of guidance, recommendations, and examples intended to support developers in creating accessible, inclusive, and user-centered digital technologies. Toolkits offer a potential means of translating insights from rigorous co-design research into practical resources that can be applied within real-world development workflows. However, concerns have been raised that toolkits risk becoming tokenistic if they are not grounded in robust evidence or evaluated in applied settings. A review of 83 health-related toolkits found that only 31 (37%) had undergone any form of evaluation, and those that had were often assessed in artificial contexts with designers present, introducing potential bias [29]. The present study sought to address this.

### Primary Objective

The primary objective of the Generating Older Active Lives Digitally (GOALD) project was to work with a diverse range of older adults to better understand their preferences and requirements in digital technologies aimed at increasing physical activity.

### Secondary Objectives

Secondary objectives were (1) to use the recommendations emerging from working with older adults to co-design a toolkit for SMEs with a focus on digital physical activity, and (2) to evaluate the perceived usefulness of the toolkit with SMEs.

The primary contribution of this study is the development of a co-design–informed toolkit for SMEs, with the participatory process and implementation evaluation presented to support understanding of its development and perceived utility.

## Methods

### Ethical Considerations

This study received ethical approval from the Faculty of Health Ethics Committee at the University of Plymouth (project ID 3034, on November 29, 2021) and from the University of Stirling General University Ethics Committee (ref: 2021 2080 2106 on October 21, 2021). All participants provided written informed consent.

### Study Design

**Figure 1.**
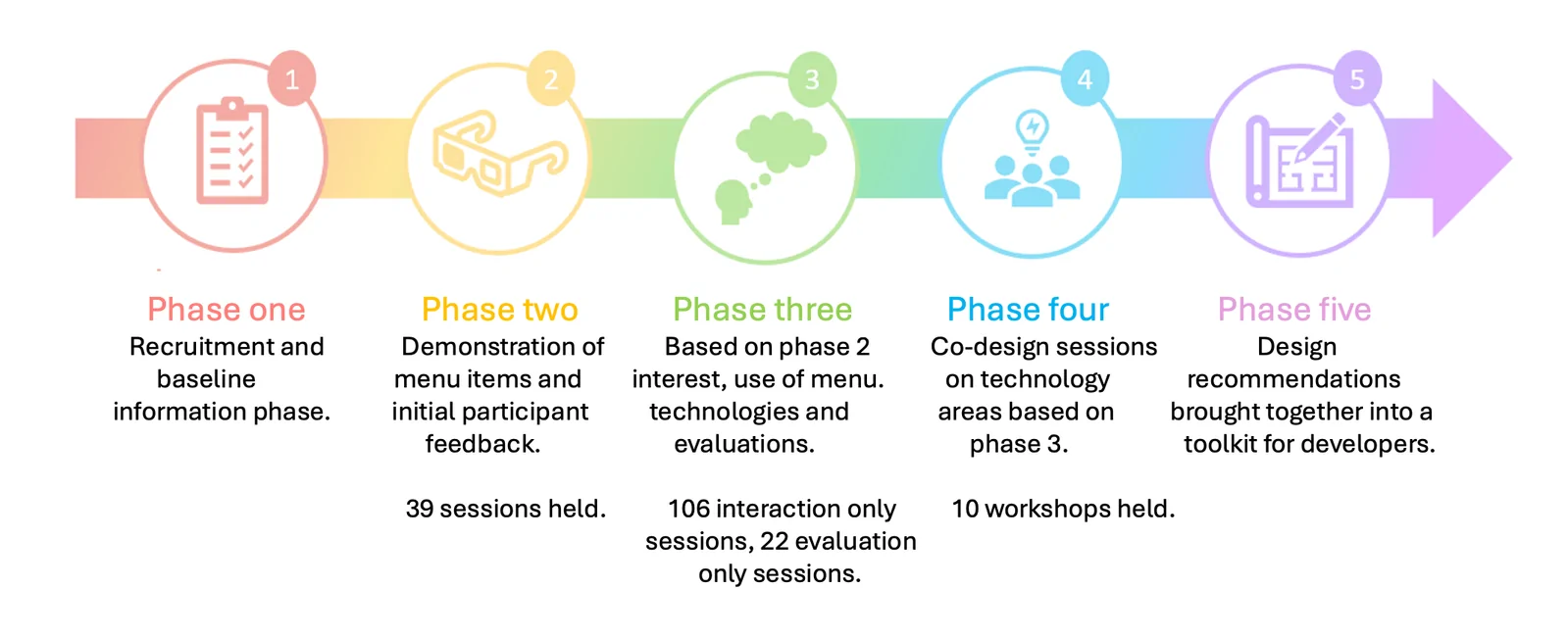
Flow diagram demonstrating the breakdown of phases of the GOALD (Generating Older Active Lives Digitally) project.

The GOALD project, a collaborative project with the aim of exploring how digital technologies can support older physically and socially active lives, was based in Plymouth (Southwest England) and Stirling (Central Scotland). Older adults were recruited via partner organizations at both sites, plus an advisory group to support the methodology. This study was split into a series of phases, as outlined in Figure 1. Phases 1‐4 involved participatory in co-design aligning with our primary objective; co-design workshops were held between June 2022 and November 2023. Phase 5 consisted of toolkit development followed by a qualitative implementation evaluation of the toolkit with SMEs, corresponding to our two secondary objectives.

Table 1 presents an overview of the purpose of each phase and details of its impact on the final toolkit.

**Table 1.** Purpose, number of participants, data collected, analysis, and impact on toolkit formation outlined for each phase and by site.

Phase	Purpose	Participants and sessions	Data collected	Analysis	Output or role in toolkit
Phase 1	Exploratory engagement and recruitment	213 participants recruited across the two sites	N/A^a^	N/A	Informed recruitment and initial engagement
Phase 2	Exploratory engagement and familiarization with digital technologies	England: 14 sessions, 40 OAs^b^, 4 YAs^c^, 9 staff; Scotland: 14 sessions, 42 OAs, 0 YAs, 11 staff	Informal feedback, baseline discussions	Not formally analyzed	Exploratory: informed selection of technologies for evaluation
Phase 3	Evaluate experiences of interacting with selected technologies	England: 27 technology interaction sessions, 22 formal evaluation sessions 60 OAs, 5 YAs, 12 staff; Scotland: 50 technology interaction sessions (with evaluation^d^), 60 OAs, 2 YAs, 18 staff^d^	Field notes, audio recordings of interactions, focus group and interview transcripts of evaluations	Reflexive thematic analysis	Direct: identified user needs, barriers, and priorities (see the study by Tomaz et al [30])
Phase 4	Co-design and prioritization of features	England: 8 sessions 28 OAs, 1 YAs, 3 staff; Scotland: 4 sessions, 18 OAs, 2 YAs, 6 staff	Ranking cards, workshop artifacts, audio recordings	Thematic synthesis + descriptive ranking	Direct: generated prioritized recommendations for toolkit, additional needs identified
Phase 5	Apply and evaluate toolkit with SMEs^e^	SMEs (n=10)	Reports, interviews	Reflexive thematic analysis	Evaluation: perceived utility and impact of toolkit. Recommended changes from SMEs implemented to produce final toolkit

a N/A: not applicable.

b OA: older adult.

c YA: younger adult.

d In Scotland, the care homes needed or wanted a lot more support than community groups or homes or groups in England with some of the more complex technologies, so some sessions in phases 1, 2, and 3 are counted twice as they included an introduction and interaction, or an interaction with a formal evaluation.

e SME: small- and medium-sized enterprise.

### Participants

Recruitment took place through direct invitation and facilitation from our partners (Active Stirling, Sports Heritage Scotland [now Memories Scotland], iSight Cornwall, Hearing Loss Cornwall, Devon and Cornwall South Asian Society, Nudge, Cornwall Rural Community Charity, Generations Working Together, and UK Active and Sporting Heritage) and local care homes. Consequently, 213 participants reflecting a diversity of age, disability, ethnicity, and location from 18 case sites initially consented to this project; participants are characterized in detail in Table 1 and elsewhere [30]. In brief, older participants’ mean age was 78.9 (SD 14.0), but the sample also included care home staff and young people aged 16‐25 years. Of the participating older adults who provided data, 63% (55/87) were living independently; 75% (65/87) reported a health condition that limited them physically, mentally, or socially, and most reported using some type of corrective or assistive aid—most commonly glasses, hearing aids, or mobility aids; and participants were based in urban and rural areas across Central Scotland and Southwest England. The overall GOALD project examined both physical activity and sports reminiscence, and some participants only engaged in one of these components.

This paper reports data collected from participants who engaged in at least one session of physical activity in phases 2‐4, resulting in 157 participants from 15 case sites across Central Scotland and Southwest England. Older adult participants were included if they had the capacity to consent, understood English, were aged 65+ years, and were living in a residential care home or attending a community group. Other participants were included if they were staff assisting the older adults in the co-design workshops due to being care staff or running the community group, or if they were members of the research team. Younger people were recruited through the community groups or from local high schools engaging in sport leadership training and intergenerational work (which was a broader goal of the GOALD project reported elsewhere [31]). However, the older adults were the main source of co-design insights, and we report the participant breakdown below simply for transparency as to who was present during the sessions. There were 80 Central Scotland participants and 77 Southwest of England participants. A total of 56 older adults were from 7 care homes and 65 older adults from 8 independent living community groups. From the staff recruited, 24 of them were care home staff with varying roles and 5 of them were facilitators from community groups. Due to recruitment difficulties, only 7 younger adults from the community were recruited in total (Table 1). Details of sites are available in Multimedia Appendix 1.

### Selection of Technology

Initial technologies were identified through the research team’s existing academic, clinical, and industry networks to enable rapid access to a diverse range of digital tools relevant to physical activity, reminiscence, and social connection in later life. As this study progressed, additional technologies were purposively identified and introduced. This was in response to older adults’ feedback and expressed interests, to ensure that subsequent evaluations and co-design activities aligned with participant priorities. Collectively, the technologies spanned multiple domains and modalities, including online physical activity platforms, immersive VR experiences, and other interactive digital tools. The technologies included in this study were not intended to represent an exhaustive or systematically sampled set of digital interventions.

As a result, older adults could try up to 20 technologies at different stages of development aimed at increasing physical activity and reminiscence. These included online physical activity platforms, VR experiences, social connectivity apps, voice technologies, camera glasses, activity trackers, balance boards, tablet-based aqua-aerobics, and social robots. Due to geographical constraints, not all technologies were available to all participants. Table 2 presents the technologies selected by participants for interaction during this project, which informed the development of the GOALD toolkit.

**Table 2.** Technologies participants interacted with throughout this project.

Name	Descriptions
Digital heritage bundle	VR^a^ experience created within the University of Plymouth, allowing users to explore heritage remotely. Users are immersed in both audio and visual media, with 3D printed models of artifacts available alongside the VR.
MOTUS VR experiences	Three devices: Standing omnidirectional treadmill with VR headset that allows users to explore both 3D scanned heritage sights and computer-generated environments. Seated treadmill where they can explore computer-generated environments, with incentives to encourage movement. A static seated experience, where 360 videos are uploaded into the headset, allowing users to be immersed in footage. As part of the treadmill experiences, remote users can be connected within virtual environments and explore together regardless of distance.
Oceans Conservation Trust experience	A VR experience created by the National Marine Aquarium (Plymouth, United Kingdom) Ocean Conservation Trust. The experience includes 360-degree videos taken from dives within the aquarium’s tanks. Marine artifacts are also included to provide tactile and educational components. This experience was delivered by the Ocean Conservation Trust team.
Oculus Store apps^b^	National Geographic Explore VR Oculus app^b^: this allows physical movements, such as kayaking and walking, to explore hard-to-reach places worldwide. Brink Traveler^b^: users can walk through and learn educational information about locations worldwide.
danceSing/ danceSing Care	Online physical platforms that provide on-demand fitness classes for a range of abilities, including yoga, Pilates, barre, and singing content. There is also a live session component, allowing users to interact with others and instructors.
Mature Movers	Online platform that supports users in finding exercises suited to their needs and provides classes for those aged over 60 years to promote physical activity independence for as long as possible. A mixture of both face-to-face and online classes.
Elder Gym^b^	Online platform that provides exercise classes for older adults, including strength, endurance, balance, and flexibility. Classes are held on a social media platform for live interactions.
Wheel power^b^	Online exercise platform with chair-based exercises suitable for those with reduced mobility or wheelchair users. Live classes, alongside on-demand content.
Goodboost	Waterproof tablet that provides water-based exercises for older adults through an avatar and written instructions.
RetroRehab	Exergaming product created by Goodboost. Online platform with interactive “arcade style” exercise games to encourage movement to improve coordination, balance, and daily activities, for example, sit-to-stand repetitions.
HUR Ltd.	Balance training platform with a display touchscreen that uses the person’s movement to control avatars in a range of games to encourage pneumatic strength and balance training.
Body Boosting Bingo^b^	A game of bingo delivered by Age Scotland in which numbers are called, and for each of the possible numbers there is an associated movement to build strength or balance. The movements can be done seated or standing.

a VR: virtual reality.

b Technologies that were provided in evaluation and co-design workshops only and not the earlier “menu” phase.

Between phases 3 and 4, researchers reviewed participant evaluations and conducted a horizon scan of available technologies based on their requests. Google searches using the phrases “virtual reality to see places for older people” and “online physical activity platforms for older adults” were conducted, with the first 5 pages of results reviewed to identify technologies aligned with participant feedback. Based on availability and location, additional technologies (also shown in Table 2) were provided for participants to trial before phase 4 co-design workshops.

### Data Collection

Data were collected across phases 3‐5 using multiple qualitative methods; earlier phases (phases 1‐2) focused on exploratory engagement and familiarization where participants were shown a general menu of technologies related to encouraging physical activity, reminiscence, social connectivity, and access to places and spaces. These phases did not generate formally analyzed data (Table 1).

Regarding phase 3, based on interest expressed in phase 2, researchers (LC, HB, RB, CBS, and SAT) attended community group sessions or visited care homes to support participants in interacting with the technologies. At Plymouth sites, all community participants received logins to access online physical activity platforms at home or during community sessions, and care-home activity leaders received logins to facilitate sessions at their convenience. At Central Scotland sites, sessions were facilitated in-person by researchers; some of these took place in the university laboratory or swimming pool. During phase 3, data included observational field notes recorded by researchers, audio recordings of participant comments, and whiteboard notes captured during group activities. Formal evaluation focus groups and semistructured interviews were conducted in person or online to explore perceived benefits, barriers, usability, and suitability of the technologies. These sessions were audio recorded and transcribed verbatim.

Regarding phase 4, co-design workshops were conducted to identify participant priorities for the technologies. Based on phase 3 findings, 3 co-design themes were addressed: (1) VR for older adults to support physical activity and access to places and spaces; (2) online physical activity platforms for older adults; and (3) general physical activity technologies for older adults. Participants were reminded of the technologies encountered during this project and completed two activities: ranking cards and idea generation.

For the ranking-card activity, researchers synthesized features raised during initial interactions and formal evaluations, categorized them (eg, usability and interaction type), and produced cards for prioritization. Participants ranked cards from low to high priority within assigned categories (one or two per group). Photographs of the ranked cards were taken to generate quantitative priority data. For the idea-generation activity, participants worked in groups to respond to the prompt: *“*If there were no limitations, what would you want from VR technologies, online physical activity platforms, or physical activity devices to motivate physical activity for older adults or intergenerational groups?” Drawing on Harrington et al [32], participants were encouraged to consider activity type, content, appearance, motivation, and practical considerations. Groups discussed, wrote, and drew ideas using large paper, pens, and sticky notes, then presented them to the wider group. This card-ranking approach was selected because it is widely used in co-design to identify together what is most important and help move beyond discussions to actionable decision-making [33]. It also provides a visible, tangible record of how decisions were reached [34]. During these phase 4 co-design workshops, data comprised audio recordings of group discussions and presentations, written and drawn artifacts produced during idea generation activities, and photographs of ranked cards generated during prioritization exercises. All recordings were transcribed verbatim.

Regarding phase 5, findings from phase 3 interaction and evaluation sessions and phase 4 activities were synthesized into preliminary toolkits to support SMEs designing technologies for older adults, with a focus on physical and cognitive activity. The toolkits were distributed to 22 UK-based software SMEs across 2 calls (July and September 2023). SMEs were invited to submit small projects to apply the toolkit recommendations to improve existing products or develop new ones; 10 SMEs were selected for funding by a panel of GOALD researchers. As a grant condition, funded SMEs provided a short end-of-project report (maximum 6 months) detailing achievements, toolkit use, challenges, observed or anticipated impact, and recommendations for improving the draft toolkits. Following receipt of the reports, researchers (LC, HB, CBS, and John Ritchie, PhD) conducted 30-minute video calls with representatives from the 10 funded SMEs to discuss their feedback in more depth, emphasizing that candid reflections were encouraged given that funding had already been awarded. One interview (Football Memories Scotland) was conducted face-to-face during a researcher visit. Phase 5 data therefore included written end-of-project reports submitted by participating SMEs and audio-recorded follow-up interviews exploring toolkit use, perceived influence on design decisions, and implementation challenges, which were transcribed verbatim.

### Data Analysis

#### Qualitative Analysis

Transcriptions from phases 3 and 4 were analyzed using reflexive thematic analysis following procedures outlined by Braun and Clarke [35]. Researchers (LC, HB, CBS, and SAT) first familiarized themselves with the data through repeated reading of transcripts and field notes. Initial codes were then generated inductively across the dataset, capturing features relevant to older adults’ experiences, preferences, and priorities. Codes were subsequently reviewed and organized into candidate themes, which were refined through iterative discussion within the research team to ensure coherence, internal consistency, and distinction between themes. Themes were then defined and named, and illustrative data extracts were identified to support interpretation. Analyses were initially conducted separately at each site due to differences in data collection timelines. These were then compared across datasets and integrated through team discussion. Where themes were conceptually similar, they were merged into cross-cutting themes. Where differences arose, these were retained and discussed within the research team to preserve contextual variation and ensure that divergent perspectives informed the final interpretation and toolkit recommendations. Analysis was conducted reflexively, with themes treated as interpretative patterns developed through engagement with the data rather than as fixed or exhaustive representations. These themes and codes formed the recommendations and produced 4 preliminary toolkits, which focused on VR for physical activity and accessing places and spaces (Plymouth), online physical activity platforms (Plymouth), general physical activity platforms including online platforms (Stirling), and a reminiscence-based toolkit (Plymouth initially, updated with input from Stirling data for the second funding call to technology SMEs).

Final reports and interviews from phase 5 challenge-fund software SMEs were thematically analyzed following the same reflexive approach. Researchers (LC, HB, and CBS) familiarized themselves with all texts before independently conducting inductive coding on sections of the data. Texts were then swapped to compare and agree on coding interpretations. Patterns in the codes were identified to create overarching themes, which were reviewed and finalized across all texts. These themes and codes were subsequently merged and refined to produce a single, consolidated toolkit combining data from both sites.

#### Exploratory Quantitative Analysis

Card-ranking results from phase 4 were calculated after rebalancing scores to account for variation in group size and number of groups per session, and results were banded into tertiles. These 3 bands indicated features ranked as higher or lower priority, with the middle band reflecting neutral importance. Scores were rebalanced by weighting each site’s feature score by the number of contributing groups and participants per group, reducing the influence of single participants or small groups. Final feature scores were averaged, excluding zero votes where features were not ranked by a group.

## Results

### Overview

From the thematic analysis of older adults’ data, 7 themes emerged: motivation, content, barriers, design/inclusivity considerations, potential audiences/suitability, acceptability of technology, and motivations to use. Table 3 summarizes the core themes that emerged across the qualitative analysis and provides an overview of the cross-cutting concepts that structure the results presented below.

These themes were separated into 3 sections for the preliminary toolkits, to ensure the information was categorized clearly for SMEs. The 3 sections were general recommendations for technology for older adults, recommendations specific to physical activity platforms (eg, websites, apps, and exergames), and VR recommendations for those working in immersive technologies. Several themes crossed over different subsections; themes and codes (recommendations) are summarized below.

**Table 3.** Overview of the major themes that emerged across the qualitative analysis.

Major theme	What it captures
Motivation	Factors encouraging engagement, enjoyment, and sustained use
Barriers	Physical, psychological, and contextual challenges to uptake
Design and inclusivity	Hardware, software, and interaction features enabling accessibility
Content	Preferred types, qualities, and relevance of experiences
Acceptability	Comfort, trust, perceived value, and social acceptability
Suitability and context	Appropriate settings, user groups, and use conditions
Motivations to use	Perceived benefits (eg, well-being, social connection, activity)

### General Recommendations for Technology for Older Adults

Themes and related codes that produced the recommendations for general technology for older adults and the evidence (quotes from participants) supporting the development of these themes are shown in Multimedia Appendix 2. Themes within this section included motivation, content, barriers, design, and inclusivity considerations. Recommendations covered suggestions from users on how to make the products motivating to use, from providing social components to incorporating gamification principles. General content suggestions included intensity of exercises, including relatable content, and that having variety and choice were key. Participants discussed barriers such as concerns regarding injury and how accessible the technology was. Finally, the theme of design or inclusivity considerations emerged, which included software considerations, such as using websites or apps, ensuring there are physical adaptations to the technology to make it easier for older adults to use, and seeking to understand what is “easy” for their target audiences to use.

### Recommendations Specific to Physical Activity Platforms

Themes and codes that were specific to physical activity platforms and supporting evidence are presented in Multimedia Appendix 3. The main themes that were specific to physical activity platforms included motivation, design or inclusivity considerations, barriers, and potential audiences or suitability. To encourage motivation in users, physical activity platforms should have engaging instructors who are relatable to their audience, such as using older adult instructors. Live sessions were also highlighted as a motivation for users, as these allowed for social interaction components. Barriers included motivating users through a screen, when many preferred going outside or engaging in face-to-face classes, and if there were live classes, a preference was for these to be away from social media platforms. Design and inclusivity considerations included having clear visual follow-along, clear instructions, and having the ability to view on large screens. Potential audiences the older adults felt these platforms would be suited to included care home residents, community groups, people living alongside those less able or independent, and those living without access to the outdoors. More detail on recommendations specific to physical activity technologies is reported elsewhere [30].

### Recommendations Specific to VR Technologies

Of the main themes, 6 were linked to VR-specific recommendations and are presented in Multimedia Appendix 4. Older adults expressed praise for the technology and enjoyed the experiences and felt safe. However, they highlighted the need for consideration of how the technology is introduced to older adults and to consider generational differences with technology use. Barriers included older adults’ attitudes to the technology and expectations such as fear of enclosed spaces and the need to consider balance and coordination when using VR technologies, alongside potential mobility challenges that impact older adults. Older adults wanted content not to be repetitive, and to avoid infantilization or child-like games. Content should be smooth, and any movements controlled. They did express concerns around addiction to technology, as well as concerns around confusion, disconnection between senses, and disorientation. Alongside psychological effects, there were concerns of physical effects such as body aches or health and safety concerns around falling or epilepsy. Older adults reported the technology must offer more than what is already available on other technologies.

Design considerations included the hardware used, such as using comfortable headsets but needing to factor in hygiene of the equipment and storage restrictions of locations. Software should include clear tutorials and guidance, have easy navigation between content, have clear and effective audio, simplified setup, and have consistent and reliable technology. Older adults raised having gamification, motivational rewards such as milestones and checkpoints within the virtual environments to encourage continued use. They also wished to be able to interact with the scenery and have movement as an option to explore, such as walking, skiing, seated movements, natural movements, rowing, and more. However, participants reported avoiding using teleportation movement in VR or any movement that results in a disconnect between senses. Participants suggested the ability to project content onto larger screens so that it becomes a group activity for more people to enjoy and facilitate communication.

Content suggestions included nature such as gardens, animals, coastal content, and under the sea. Historical content was also requested, including wonders of the world, archaeology, National Trust sites, museums, and archive footage. A range of activities were raised, such as experience sports, journeys on different modes of transport, shopping, visiting new locations, guided tours, and wanting a mix of passive and active experiences. Alongside this, there was a desire for personal content that could link them to familiar places or past holidays. Overall, older adults wanted longer content, colorful scenery with soothing audio, realistic imagery with details, audio guides, educational content, and most importantly, variety.

Older adults reported various perceived benefits that motivated them to use VR devices. One motivation to use VR was the enhanced experiences it could offer, such as giving access to places that older adults cannot access, whether due to mobility, cost, or mental health challenges, or activities that would otherwise be unsafe. VR offered them the opportunity for new experiences and to see new places, as well as providing an educational angle. As well as new experiences, the devices could be used to escape reality or relive old memories, so it would provide mental and physical stimulation. The physical motivations were the ability for it to facilitate exercise and provide interesting and distracting content while doing so, and identified the ability for it to have therapeutic uses. Older adults expressed the fun and enjoyment they had from using VR, and that it had potential to offer a social experience.

They felt that VR technology that increased physical activity and gave access to places and spaces was suitable for care home residents, people who needed more exercise, those with mobility issues, or those experiencing pain. It was highlighted that the technology could be suited to those who require rehabilitation, or those who are hospitalized or bed bound.

### End-User Priorities for Technology SMEs From Rankings

Results from the exploratory ranking analysis in the co-design workshops where participants ranked their priorities for development are presented in Tables 4 and 5. These rankings should be interpreted as indicative outputs of workshop-based prioritization rather than generalizable or statistically robust measures.

**Table 4.** End-user priorities ranked for physical activity platforms for older adults.

Priority level	Components
High priority	Follow-along videos, community of users (hosted in the platform), group activity in-person, visual follow-on, variety of content, live sessions, filter function on difficulty level, content for range of abilities, variety of songs to move to, assessment on suitable exercises, voice assistive technology, game scenario—reaching, stretching, moving, armchair activities, warm up and cool down activity, easy to navigate, ability to pause and come back, value for money (affordable), motivation to exercise, easy to use, easy to search, audio instructions (sight impairment), easy initial setup (sight impairment)
Medium priority	Gamified component—collecting badges, easy screen casting options, indoor cycling, varied cultural content, separate from social media platforms, audio description to navigate platform (sight impairment), gradual increase in intensity, individual activity, social forum component, yoga, music content for varied generations, someone to check your exercises, family activity, adjustable pace of sessions, adaptable video and font sizing, older tutors to match audience, big screen or not a mobile app, appropriate length (<45 min), translation options, closed captioning, frequent updates, space needed for activities, clear audio (speaking over music), dancing, group activity, range of video lengths
Low priority	Tutorial to navigate platform, extreme sports (skiing), reminders to take part (notifications), needs to be different from what is already received for free

**Table 5.** End user priorities ranked for VR^a^ technologies for older adults.

Priority level	Components
High priority	Pedaling, people watching, guided tour, audio from the scenes filmed, different eras, varied content, multisensory (artifacts to hold, breeze to feel, and sand at beach), simple setup, historical information on audio, armchair exercise, information on what you are viewing, relaxing visual content, colorful, bright, interaction with objects, clear instructions on how to use, natural foot movements (walking not shuffling), places with memories, safety features on exercise equipment, comfortable headsets, natural movements, places usually expensive to visit, movement with feet, ability to move around in virtual environment, headset not too heavy, new experiences, not colorless, places no longer physically accessible, realistic, affordable price, detailed visuals (fine details, eg, leaves on trees and petals), cleanable, not simplistic models, high image quality
Medium priority	Rewards (incentives to continue [seeing something new or exciting]), show you locations you may have to go (reduce anxiety hospital appointments and shopping trips), pretravel knowledge (view where you will go), group activity on a big screen displaying VR content, POV^b^ adrenaline experiences (zip lining, roller coasters, and horse riding), not cartoonish, physical activity associated with hobbies, that is, gardening, audio, sounds, or music (eg, blue planet), watch animals, sports matches, social element should be optional, seated movements (technology allows seated interaction), passive watching of nature, archeology, inclusive (mobility), related to hobbies, local places, dancing, family interaction, warnings on content (small spaces, heights, sit down, or dizziness), pedals to move around, menu self-orientates in front of you, outdoors, seaside, flowers, heritage sites, movement with hands, haptic feedback technology (gloves), historical events (jubilee or reminiscence), step counter, walking, stable movements (smooth and not teleporting), has to offer more than television, feel control over moving, subtitles (for hearing impairment), headphones for sounds, easy to put on headset, different countries, written cues to sound (for hearing impairment), concerts, interesting content, nature, countryside, educational aspect, paddling or rowing, audio journey or description (for sight impairment)
Low priority	Shopping trips (malls, in stores you no longer go to), treasure hunt (items to find), garden centers, races, social VR world, transferable or portable, peaceful places, busy environments (more hustle and bustle), competitions (including with family or grandchildren), races, audible directions of where to go (sight impairment), avatar customizable, historical information plaques, calm sounds, directional seated movement

a VR: virtual reality.

b POV: point of view.

**Figure 2.**
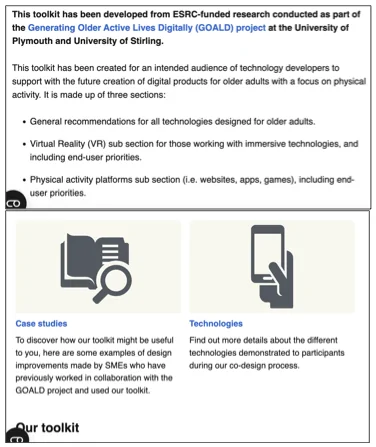
Screenshots of the toolkit to exemplify its contents. ESRC: Economic and Social Research Council; GOALD: Generating Older Active Lives Digitally; SME: small- and medium-sized enterprise; VR: virtual reality.

Features were separated into 3 priority categories based on clustering the features according to calculated priorities to offer SMEs guidance on which features to prioritize in their product developments. The priorities were calculated by balancing the ranking across the venues, workshops, and group sizes to equal out scores from individuals over scores from groups. These were provided to the SMEs who won the challenge fund competition alongside the preliminary toolkits. The full GOALD toolkit is available online [36]. Screenshots illustrating the toolkit are shown in Figure 2.

With the support of the £5000 grants (a currency exchange rate of £1=US $1.23 was applicable) and use of the toolkits, 10 SMEs (Multimedia Appendix 5) were each awarded £5000 based on their ideas to use the preliminary toolkits and the likely impact of the product. They made meaningful adaptations to their products, enhancing accessibility, user experience, and engagement. Changes included merging platforms for better navigation, adding customization and accessibility features such as subtitles, creating instructional guides and videos, integrating physical activity trackers, improving content variety, and developing voice-based and intergenerational features. Several SMEs also introduced new software modules, games, and apps, addressed safety and inclusivity, and tailored content to better suit the needs and preferences of diverse user groups (Table 6). Physical activity-specific adaptations are considered and reported elsewhere [15,16,30].

**Table 6.** Adaptations made to products as a result of the challenge fund and toolkits.

Business	Adaptations with challenge fund
Mature Movers	Updated content to have varied workout duration and more variety, took into consideration instructor preferences, added sections and filters, and began the process of exploring recommended exercises.
Triangular Pixels	Added physical activity tracker into virtual reality game for feedback and motivation.
Sentai	Added intergenerational connectivity and reminiscence feature and added voice-based physical activity encouragement.
MOTUS Ltd	Improved set-up procedures and created instructional videos to reduce reliance on the MOTUS staff facilitating sessions.
danceSing	Merged 2 websites into a single platform, added more intuitive navigation, added more user customization options, added accessibility features, for example, subtitles, added instructional guides on using the platform, moved live sessions to the website instead of social media, added a user chat function, added content filters, added analytics on user engagement, used age-relatable instructors.
Age Scotland	Enhanced accessibility features, added subtitles, improved warm-up and cool-down videos, added relatable music and older models, added direction on breathing and posture, explored ways of supporting with safety, added software for alternative languages, added different intensities of exercises.
Good Boost	Included new games into the retro-rehabilitation game that focus on strength, balance, and coordination, developed aquatic resistance exercise equipment to help with strength.
HiCarer	Built a module within the app to support people in finding virtual physical activities and added filters for the content, added activities with music and seated movements based on toolkit recommendations.
Weekday Wow Factor	Created Amazon Alexa voice app to provide people with information on group activities, addressed accessibility concerns, made the app multimodal (audio and visual prompts).
Football Memories Scotland	Created audio and visual content for reminiscence and mental activity.

### Themes From Analysis of SME Toolkit Users’ Reports and Interviews

From the SME reports and interviews, 6 themes emerged. There were 4 themes related to the impact of using the toolkits on their own product development: adaptations, business benefit, enhanced end-user insight, and challenges to development. The SMEs were also asked for feedback on the toolkits themselves so the final version of the toolkit could integrate their feedback. Themes relating to the toolkit and its further development were aspects of the toolkit positively received, and improvements to the toolkit.

SMEs used the toolkits’ suggestions to adapt their products, such as training older adults as instructors, improving exercise delivery, offering varied workout lengths, adding website filters for easier navigation, and simplifying VR setups. They also introduced activity trackers, new delivery platforms (eg, Alexa widgets), and AI-voice features to motivate users. Respondents said the toolkits provided valuable context and helped develop their ideas further. The SMEs reported business benefits, that using the toolkits boosted their credibility, confidence, and speed in making user-focused design decisions. It was especially valuable for early-stage product development, helping expand their audience and market potential. SMEs noted increased enthusiasm, higher product engagement, and reduced support needs due to usability improvements. Economic benefits included easier sales, increased or projected increased sales, lower operational costs, and higher product uptake. The toolkit also supported investment by showing alignment with user needs, and the GOALD challenge fund helped accelerate funding and product changes.

The toolkits provided valuable, enhanced end-user insights, helping SMEs adjust priorities, validate decisions, and inspire new product ideas while avoiding poor design. It increased their audience awareness and highlighted the importance of co-design, encouraging further user involvement. SMEs saw the toolkits as a lasting resource they could keep using and expanding with their own findings. SMEs also reported challenges including development costs, limited funding aligned with older adults’ needs, and difficulties in conducting research and measuring impact. They also faced obstacles integrating into certain settings, accessing community facilities, and managing time constraints or technical delays.

Regarding the toolkits themselves, the positive feedback theme highlighted the ranking features for helping prioritize developments, supported by the broad recommendations. Overall, SMEs reported finding the toolkit simple, concise, and easy to follow with clear step-by-step guidance. They suggested improving the toolkit by adding more guidance on co-design research, explaining the recommendations’ background, merging toolkits to reduce repetition, adding visuals, hosting it online, and including interactive features such as checklists and case studies. They also recommended showing participants a wider range of technologies to avoid bias. Some SMEs felt the toolkit’s usefulness varied depending on the developer’s experience and stage in their journey. To illustrate the utility of the toolkits, quotes illustrating themes and codes are presented in Multimedia Appendices 6 and 7.

## Discussion

### Principal Findings

This study primarily reports the development of a co-design–informed toolkit for SMEs, supported by a participatory process and qualitative evaluation of its perceived utility.

The GOALD project engaged older and younger adults in intergenerational co-design to produce recommendations for developers, particularly SMEs, designing technologies for older adults, with a focus on increasing physical activity and reminiscence. This process resulted in a final toolkit providing developers with insight into older adults’ priorities. Consistent with previous research, older adults perceived benefits of digital health technologies for healthy aging, including physical activity, and identified diverse potential deployment settings [16]. Challenges using some technologies independently or within care settings reinforced the importance of co-designing accessible technologies [18]. Despite these challenges, familiarization enabled older adults to provide usability solutions. Preliminary toolkits and evaluations suggested that this approach could help software developers, particularly SMEs, by reducing the time and cost of considering older adults’ needs, while supporting conversations with investors.

Older adults engaged in using the technologies and evaluating them. The benefits of familiarizing themselves with the technologies to guide their suggestions in the final co-design workshops were evident in the abundance of suggestions provided by participants, as others have demonstrated [32]. Interest was, however, uneven across technology types, resulting in the toolkit focusing primarily on VR and online physical activity platforms. To increase applicability, a general technology recommendation section was included to capture codes relevant across multiple technology types. Although exposure to all possible technologies was not feasible, the toolkit is designed to be updated as new co-design research and technologies emerge.

This study suggests that older adults can successfully engage in co-design workshops; however, it was important to adapt to their needs and preferences to ensure they were able to fully participate in the sessions. This included providing technologies and researcher support to connect to online sessions, such as large screens and microphones to assist with hearing. Others have highlighted how not adapting to the older person’s needs can result in resistance to participate in co-design and a loss of the credibility surrounding co-design [37]. Therefore, the GOALD project worked with an advisory group to guide the design of the current project and, during workshops, gave longer allocated time for sessions, moved closer to support with hearing, and built rapport with the participants to adapt to their requirements. Many participants became familiar enough with VR headsets to suggest content, often focused on reminiscence, supporting the value of such content for well-being [30]. VR SMEs may particularly benefit from the GOALD toolkit for future older adult projects.

Addressing prior research limitations, which focused on older adults with higher income, education, and digital competence [38], GOALD recruited participants from diverse backgrounds, including care home residents and independently living older adults with physical, visual, and hearing impairments. Recruitment of younger adults for intergenerational co-design was more limited, due to COVID-19 and scheduling conflicts, despite evidence of mutual benefits such as improved attitudes toward older adults and increased community connectedness [39]. Future research should emphasize and communicate these benefits to younger participants.

Dissemination of co-design findings often omits end users, reducing uptake of feedback [40]. GOALD’s evaluation of the toolkit with SMEs indicated its perceived value in helping them address older adults’ needs, with updated versions available [36]. Unlike previous guidelines for older adult technology design [41], which were generic, this toolkit offers specific, participant-informed guidance. Systematic reviews have noted the merits of older adult involvement in design but often lack evidence on real-world viability or acceptability of resulting technologies [42]. The present study contributes to addressing this gap in knowledge. Further, 4 of the 10 SMEs had not had their software or devices evaluated in our co-design workshops, suggesting transferability of the toolkit ideas beyond the products on which they were based. Many publications or toolkits offering guidance on software design are very “generic” such that software developers might have guessed the advice, for example, “easy navigation” and “good user interface.” This research has produced guidance that is more specific, strengthened by examples from older participants and the target market. Previous co-design studies have used structured usability and acceptability evaluations to assess older adults’ willingness to interact with specific technologies, such as social or assistive robots [43,44]. These studies typically focus on the feasibility, usability, or acceptability of a single system within a defined evaluation context. In contrast, this study adopted an upstream, design-focused approach aimed at generating transferable insights and recommendations across multiple digital technologies and real-world SME development contexts. These approaches are complementary, with structured usability and acceptability evaluation representing an important subsequent step following toolkit-informed design. This distinction highlights that toolkit-based approaches operate upstream in the design process, whereas usability and feasibility studies typically evaluate downstream implementation and user interaction.

While participating SMEs found the toolkit useful and were able to apply its recommendations, embedding it into routine development practices over the longer term remains challenging. Ongoing use is likely to depend on practical factors such as time, resources, competing priorities, staff changes, and the stage of product development. These factors help explain why SMEs applied the toolkit selectively in this study and suggest that future evaluations should examine how well such resources fit within everyday SME development practices over time.

Future research should evaluate the toolkit prospectively by embedding it from the earliest stages of product or service development and following its use over longer timeframes. This study suggests that the toolkit informed both adaptations to existing products and the development of new features and services. However, a fully prospective “from inception” evaluation would enable assessment of how toolkit recommendations shape design decisions across the full development lifecycle and their downstream impact on end-user outcomes. Such studies would benefit from longer funding periods and closer integration with SME development cycles to support sustained implementation and evaluation.

### Limitations

This study has several limitations. Feature priorities were ranked to indicate their relative importance to older adults; however, differing timelines across sites meant that features could not be merged into a single ranking exercise. This resulted in separate initial toolkits for England and Scotland. In addition, not all technologies were available at every site, and facilitation differed across settings, which may have influenced participants’ experiences and priorities. These differences across settings are discussed in detail elsewhere [30].

The card ranking exercise used in phase 4 was intended as a pragmatic prioritization tool to support co-design rather than as a validated quantitative measure. Rankings were used to indicate relative importance of features within the workshop context, and although steps were taken to rebalance scores for variation in group size and participation, the approach remains exploratory and context-specific. As such, ranking outputs should be interpreted as indicative of older adults’ priorities to inform toolkit development, rather than as definitive or statistically robust measures of importance across populations or contexts. Despite this, participating SMEs reported that the toolkits provided specific, practical guidance that could be directly implemented to update or adapt their products.

The evaluation of toolkit impact was, of course, based on self-reported feedback from participating SMEs rather than the direct measurement of downstream design processes or end-user outcomes. This approach was taken because this project’s funding window and scope did not permit longitudinal follow-up or independent assessment of postimplementation outcomes within participating organizations. As such, findings reflect SMEs’ perceived utility and influence of the toolkit on their decision-making, rather than objectively measured changes in product quality or user outcomes. Reported impacts may therefore be subject to social desirability or recall bias, although this risk is reduced by the fact that feedback was collected after funding had been awarded and toolkit use had occurred. In addition, adoption of the toolkit recommendations varied across organizations, reflecting differences in development stage, resources, and priorities. This heterogeneous and partial adoption limits generalizability but reflects realistic patterns of toolkit use within SME development contexts.

The provision of the small challenge funds was likely to have been a catalyst for the use and success of the toolkits, but this was a crucial first step in testing them as part of the GOALD project. Future research could assess the resulting impact on older adults of adaptations to technologies made based on the toolkits; this was beyond the scope of the present research. Finally, the toolkits were produced following the selection of a limited range of digital products focusing on keeping older adults active. Nevertheless, 2 of the 10 SMEs awarded funding were from other areas (HiCarer, a directory type application, and Sentai focusing on connecting people with friends and family), suggesting broader applicability of the recommendations.

### Conclusions and Future Research

Involving older adults in the co-design of technologies to support healthy aging is important for developing technologies that are more likely to be acceptable and relevant to older adults.

The GOALD project engaged older adults in co-design for technologies to support healthy aging with a focus on aiming to increase physical activity and connectedness to produce the present toolkit. This study focused on upstream design and implementation processes rather than direct measurement of aging-related health or functional outcomes. However, the toolkit was intended to support age-appropriate design decisions that may influence downstream usability, engagement, and outcomes. Evaluation of health, functional, or behavioral impacts requires longer-term implementation and outcome-focused study designs and is therefore an important next step for future research. The toolkit may act as a knowledge exchange between the research with older adults and with current and future SMEs, to help facilitate continued developments and engagement with co-design. SMEs who tested the toolkit reported useful content which informed some of their product adaptations and was perceived to support some business benefits. Future research could consider evaluating the impact of toolkit-informed adaptations to technology on older adults. It could also incorporate the results of co-design work on a broader range of technologies designed to increase other activity beyond physical activity and reminiscence.

## Supplementary material

10.2196/89785 Multimedia Appendix 1 Descriptor of case sites across England and Scotland (including breakdown of participants at each site).

10.2196/89785 Multimedia Appendix 2 Main themes and codes, with evidence, used to form the general recommendations for technology for older adults (case site numbers provided).

10.2196/89785 Multimedia Appendix 3 Themes and codes, with evidence, that formed the recommendations directed toward physical activity platforms (case site numbers provided).

10.2196/89785 Multimedia Appendix 4 Main themes and codes, with evidence, used to form the recommendations for virtual reality for older adults with the focus on increasing physical activity (case sites provided).

10.2196/89785 Multimedia Appendix 5 Organizations awarded funding with project aim and hoped-for outcome.

10.2196/89785 Multimedia Appendix 6 Quotes from Mature Movers coded in the thematic analysis showing themes.

10.2196/89785 Multimedia Appendix 7 Quotes from developer “4” coded in the thematic analysis showing themes.

## Abbreviations

GOALD: Generating Older Active Lives Digitally
SME: small- and medium-sized enterprise
VR: virtual reality
