# Supplementary material for: Developing Digital Platforms to Help Older Adults Stay Physically and Socially Active: Co-Design Study to Develop a Toolkit and Evaluate Its Impact
Source: JMIR Aging. 2026 Aug 27;9:e89785. doi: 10.2196/89785 (PMC13521207; doi:10.2196/89785)
Supplement: Supplementary file 1 [file aging-v9-e89785-s001.docx]

| **ID** | **Type** | **Descriptor** |
| --- | --- | --- |
| CS1 | Care home (England) | Residential home providing nursing, residential, dementia, respite, intermediate and palliative care. (7 older adults, 3 staff). |
| CS2 | Care home (England) | Residential care home providing respite care, specialising in dementia and palliative care. (10 older adults, 2 staff) |
| CS3 | Care home (England) | Residential and nursing care home providing respite, nursing, and palliative care. (1 staff) |
| CS4 | Care home (England) | Residential care home providing nursing, respite, and dementia care. (4 older adults, 2 staff) |
| CS5 | Independent living (England) | Community group organisation dedicated to supporting and strengthening Cornwall’s Communities to improve quality of life. Group was a technology support group for the local community. (18 older adults, 1 young adult, 2 staff) |
| CS6 | Independent living (England) | Community group with a mission to build a strong local community and economy. (6 older adults, 1 young adult, 1 staff) |
| CS7 | Independent living (England) | Community group with the aim to create a cultural, social, and mutual support network for the local South Asian community. (1 older adult, 1 young adult) |
| CS8 | Independent living (England) | Charity group promoting inclusion and deaf awareness in the region, aiming to create stronger links for the d/Deaf community. (5 older adults, 1 young adult, 1 staff) |
| CS9 | Independent living (England) | Charity group supporting anyone living with a visual impairment, with the aim to afford the visually impaired support and encouragement to lead active, independent, and involved lives. (8 older adults) |
| C10 | Care home (Scotland) | Residential care home providing nursing and respite care. (10 older adults, 4 staff) |
| CS11 | Care home (Scotland) | Residential care home providing nursing and respite care. (4 older adults, 4 staff) |
| CS12 | Independent living (Scotland) | Participants were from a community falls and balance group. The class is delivered by a local leisure provider. (19 older adults, |
| CS13 | Independent living (Scotland) | Participants were from a community falls and balance group. The class is delivered by a local leisure provider. (7 older adults) |
| CS14 | Care home (Scotland) | Residential care home providing nursing and respite care. (11 older adults, 5 staff) |
| CS15 | Independent living (Scotland) | Participants were attendees of a local community hub. The community hub delivers activities which have a social element to bring the community together and aims improve the lives of people with disabilities; promoting inclusion and equality for the young and elderly; tackling social isolation and health and wellbeing; and connecting the local community. (4 older adults, 2 staff) |
