# Supplementary material for: Developing Digital Platforms to Help Older Adults Stay Physically and Socially Active: Co-Design Study to Develop a Toolkit and Evaluate Its Impact
Source: JMIR Aging. 2026 Aug 27;9:e89785. doi: 10.2196/89785 (PMC13521207; doi:10.2196/89785)
Supplement: Supplementary file 2 [file aging-v9-e89785-s002.docx]

| **Theme** | **Code/ recommendation** | **Evidence** |
| --- | --- | --- |
| Motivation | Social connection and fun | average about 10 residents in there at a time[...]and then that is something that they can all engage in. […]they are entertained, they are being stimulated, (CS3) |
|  | Gamification Principles | But again, I quite like, with the goals and stuff, the target. If you put games on it, like try to get to a certain point, or race against someone, (CS7) |
|  | Benefits of technology/product | I think psychologically it’s absolutely massive. I've got relatives at the moment in care homes and they're not stimulated at all. […]. They are literally just sitting there. And I know we’re short of staff in every arena at the moment now, but if they just had access to something like this where they could get a few minutes a day, I just think that would make the hugest difference, just to their psychology. (CS8) |
| Content | Type of activity | So, I would say at the moment, there is limited things available. […] So, there wasn’t a vast amount of content on there. […] But yes, it would be nice to have a bigger variety, more songs, different genres. (CS3) |
|  | Relatable content for engagement | avoid modern music - needs to be generation appropriate (CS6) |
|  | Intensity | And even if it might not be like the hardest exercise for younger people, maybe if they had two separate levels, […]they do an easy version of an exercise or a more difficult version of an exercise maybe that could be useful. (CS7) |
|  | Duration | But it’s all exercises, you do it, it doesn't matter whether they’re 20 minutes, 30 minutes[…]. You can slowly build up from 5 minutes and 10 minutes and 20, 30 minutes. (CS7) |
|  | Variety of content | You’ve got to have change otherwise you’ll get all just one stuff all the time, so you need change. (CS1) |
| Barriers | Injury concerns | You’ve got to be a bit careful with yoga. If you’re not used to it and you overdo it, it can take months and months for your muscles to repair themselves. (CS6) |
|  | Accessibility (physical capabilities) | Yes. I've got a tremor and very often I press the buttons and it automatically presses it the second time which doesn’t help the situation. (CS8) |
|  | Accessibility (technology) | I’m just thinking, because I’m reasonably okay technically and I can use the programmes and I can access them, and if it goes wrong I can usually sort it out. If you’re using it yourself at home, for somebody who’s not perhaps as tech savvy, it might not be as attractive. […] from the people I run a little digital inclusion hub – and I’m not sure whether many of those would be able to cope with it. (CS6) |
|  | Accessibility (space and social context) | Then you're quite restricted to the amount of space you’ve got if you're in your own home, because I could probably find somebody to set it up on the telly, but where our computer is is probably the best place. (CS8) |
| Design/ Inclusivity considerations | Infrastructure usability | If they had something that remembered us so you're not logging in each time and going through the same thing, it comes up with your home page and saved dances, suggestions for dance videos, I think that would help with ease of access. (CS8) |
|  | Tailoring/filtering | I thought they could do with a ‘limited mobility’ filter on the dropdown menu so you can filter for ‘appropriate for all’ or ‘beginner’ or ‘advanced’, but maybe having a ‘limited mobility’ there would be good. (CS6) |
|  | Suitability of hardware for environment | Supposed to be water resistant but splashes cause it to skip (CS12) |
|  | Software | Simplify setting it up. The simpler to set it up, the more people are likely to use it. (CS5) |
|  | Ease of use for target audience | I want to be able to click on what I need to do very easily. And if it’s a complicated website, you give up before you’ve even got to the activity. (CS8) |
|  | Software adaptations for physical or mental capabilities | It would be great if there were, like, subtitles that came up with that, [for hearing loss], I just think that would help them to focus a bit more because I’d be so distracted looking at things so I'm not listening out. Like there, I didn’t know that there was sound turned on. But if I had some sort of cue that there were birds tweeting. I'm just way too involved to stop moving around, just waiting to hear the birds. (CS8) |
|  | Physical adaptations of environment use | Only by turning. If I had something that was controlling it, so I could move it over here, or move it over there. But maybe the swivel chair might do that for me. (CS6) |
|  | Accessibility of exercise | But again, if it was maybe someone who can’t speak English as well, maybe that might be a difficult sort of thing. If there was a translation but then I know stuff can get mixed up in translation so I’m not completely sure if it was transferable to other communities, if you (unclear 0:21:01) speak English (CS7) |
