# Supplementary material for: Developing Digital Platforms to Help Older Adults Stay Physically and Socially Active: Co-Design Study to Develop a Toolkit and Evaluate Its Impact
Source: JMIR Aging. 2026 Aug 27;9:e89785. doi: 10.2196/89785 (PMC13521207; doi:10.2196/89785)
Supplement: Supplementary file 3 [file aging-v9-e89785-s003.docx]

| **Theme** | **Code/Recommendation** | **Evidence** |
| --- | --- | --- |
| Motivation | Engaging instructor | *I think they struggled trying to make it something, but I felt the presentation was just lacklustre (CS12)* |
|  | Live sessions | *It’s more I was getting reminders popping up saying a live is coming on now and they do them throughout the day, so it’s daily I've been getting these and they run one in the morning or evening[…]With the Facebook group, because there's reminders and you're seeing other people getting really excited about it, I think that drew me in more and made me want to go more frequently as well and get to know some of the people. (CS8)* |
|  | Instructor relatability | *If it’s aimed at older people, I think having older people doing it on the screen would be better. (CS6)* |
| Barriers | Programme design barriers | *I like face-to-face rather than screen. I find it quite difficult to keep the momentum going when I’m at home on my own and looking at the screen, and then thinking “Oh, am I doing it properly?” (CS6)* |
|  | Use of social media platforms | *And [pause] I think it would also be good to have for the social aspect, like I was saying before about the platform to talk, but not having it just on Facebook because a lot of people won’t go to an external site to talk about stuff, they will want to stay all internal, (CS6)* |
| Design/ inclusivity considerations | Clarity of presentation | *More time to see demonstrations of exercises as it can take a while to work out if you are doing it correctly (CS12)* |
|  | Clarity of instructions | *I thought the people who were talking you through it, all the tasks and exercises, I thought they were very clear and I guess that’s what I liked most about it. I felt that they were clear anyway, […], but I found it quite easy to understand them (CS7)* |
|  | Visual considerations | *I’d like to see an option to cast to the TV because a lot of things like YouTube allow you to send whatever’s on your screen straight to your TV screen without needing a HDMI cable. I found I couldn’t do that so I was digging around trying to find all the cables.[...]Usually I’d throw the screen at the TV and carry on, but they don’t seem to think about what if people want to get this up on a bigger screen and I wish they had some tool to do that and also instructions for people unfamiliar with using cables or casting, because that let us down a bit. A lot of us watch on our mobile phones or small tablets. I was zooming in and out. It was a bit time consuming to go through it all (CS8)* |
|  | Accessibility of exercises | *Also, I do get a bit bitter if a site doesn’t cater to different disabilities, especially in the modern day. There are so many people with hearing loss or vision issues. When you're catering towards the elderly or care homes, I thought it was really short-sighted. It was a bit of a stubbornness [laughs] challenges. (CS8)* |
| Potential audiences / Suitability | Care home residents, community groups, people living alone, those less able or independent, people living without access to outdoors or recreation. | *So, it would be… Something like this, where I can set it up, and I can bridge between what they are seeing and try to encourage them to do some of the movements and do some of the singing and stuff.[…]But visual, anything that is visual, or things that they can listen to, something that could be set up by someone else, but they would still be able to use. (CS3)* |
