# Supplementary material for: Developing Digital Platforms to Help Older Adults Stay Physically and Socially Active: Co-Design Study to Develop a Toolkit and Evaluate Its Impact
Source: JMIR Aging. 2026 Aug 27;9:e89785. doi: 10.2196/89785 (PMC13521207; doi:10.2196/89785)
Supplement: Supplementary file 4 [file aging-v9-e89785-s004.docx]

| **Theme** | **Code/ recommendation** | **Evidence** |
| --- | --- | --- |
| Acceptability of technology | Praise | *I absolutely loved it and I am the envy of my grandchildren. That tells you a lot, it was wonderful. Thank you (CS5).* |
|  | Facilitation | *requires a bit of time and a bit of support, to get people set up in their homes. Especially if they're not familiar with it. Like I said, even for me, I'm familiar with it, I just needed a bit of prompts, a bit of instruction, and whether that’s an instruction leaflet that can be given to people, or just a video call set up (CS8)* |
| Barriers | Attitude | *I also think there’s a certain amount of the gaming idea, in my mind, and I don’t spend any time doing that sort of thing. I don’t do any gaming, or all that on my phone. And with my grandchildren, I think of it like, you’re all here, so that was a waste of time. So even if I were in a game, I’m not sure I’d want to spend ten minutes on. I’d rather sit and knit something. (CS6)* |
|  | Mobility | *Yes, I found the stand-up one unsettling, again.[…] I found I had to get on, and moving my feet, it just got uncoordinated. My balance tends to go. Although I had hold of the rails, that wasn’t particularly reassuring. (CS6)* |
|  | Content | *Yeah, the environment’s okay, but actually there’s nothing you can do. (CS8)* |
|  | Psychological | *But for some reason or other the sea coming in, I started to disconnect with what my brain was saying and my eyes were saying. (CS5)* |
|  | Physical | *But it suddenly struck me that some people don’t have legs, or their legs don’t work anymore. So maybe there could be a system where they could do it with their hands, or even with their gaze, (CS6)* |
|  | Health and Safety | *There was no way. They would’ve fallen and then you would’ve had a massive safeguarding thing on your hands. (CS2)* |
|  | Unfulfilled purpose | *I’m just trying to think of the value of the VR over the value of watching something on television. So, just thinking about the historical events, there’s a lot of good filmage on television. And I’m not sure what the value of seeing it on VR would be. (CS6)* |
| Design considerations | Hardware | *But for those in a bigger screen, if you are doing it in a community, ten or twenty people in one go, the bigger screen may be much better in one way, that at least we’re not […] and everybody is enjoying it. (CS7)* |
|  | Software | *Maybe that’s why I didn’t actually see as much as I could have done, had I actually tilted it. I didn’t realise that I was supposed to be doing that. Yes, so instruction for people like me is quite vital. Young people seem to know what to do anyway. (CS8)* |
|  | Software for motivation | *But again, I quite like, with the goals and stuff, the target. If you put games on it, like try to get to a certain point, or race against someone, (CS7)* |
|  | Features to avoid | *they are very unnatural sort of foot movements, I think I said it before, but if you can arrange it so it’s like proper walking, rather than just sort of shuffling your feet backward and forwards. (CS6)* |
|  | Modes of interaction to consider | *But the things where you are actually physically doing things, like the one we did last time with canoeing and rock climbing, personally I found that was quite enjoyable, more so than when you are just looking round it. (VR co design)* |
| Motivations to use | Enhanced access to experiences | *I mean, especially as you're getting older, you can envisage times when you're not going to be able to get out, and to be able to visit somewhere that you've always wanted to go or go to the coast and you can’t any longer take yourself there, that would be wonderful. (CS5)* |
|  | Expanding experiences | *I've always been interested in archaeology and that type of thing, so if you were out on a dig, I would enjoy that, although I've never been on a dig, but I would enjoy seeing stuff that is being found and all that sort of thing because it’s always been an interest of mine. (CS8)* |
|  | Educational | *So, it could be used as a learning tool as well, couldn’t it? (CS5)* |
|  | Wellbeing | *Yes, and I think it would be relaxing for some people. (CS1)* |
|  | Physical activity | *it would help with improving my ability to move my legs, because it’s quite a challenge to get my legs moving. Whereas, if I had this, I don’t know, perhaps once a week or something, it would stop decline, at least. (CS6)* |
|  | Health | *I think psychologically it’s absolutely massive. I've got relatives at the moment in care homes and they're not stimulated at all. And I go and I'm, like, “I so don't want this,” and you go in there and they’ve all just got these walkers, and they're just sitting there. […] but if they just had access to something like this where they could get a few minutes a day, I just think that would make the hugest difference, just to their psychology. (CS8)* |
|  | Happiness | *And see, to me, it’s really fun. Because I love heights, I love jumping off stuff. Yes (CS6)* |
|  | Social experience | *That would be wonderful. It could be like I could be talking to my son about it and we could reminisce where we have been and what we have done and so on. (CS5)* |
| Content | Nature | *For me personally, it would be quite interesting to have a guide to the natural things, […] bits of videos of a nature reserve, where animals actually appear, and saying something about them […]The sort of experience that you couldn’t actually have unless you sat in a hide for three days in a nature reserve. […] which could then be put together for an experience, I would really like that. (CS6)* |
|  | History | *I wonder whether if you were perhaps trapped at home, or in a home, or whatever, it might be a good idea to have the idea of some of the celebrations, the sorts of things that we’ve been seeing with the Jubilee. So that it would bring back memories of things in the past. (CS6)* |
|  | Activity | *I mean, again, I can see when we were talking about somebody who can’t got to places, to then have a tour of a castle or a stately home or gardens, would be lovely. (CS12)* |
|  | Personal | *Because if it’s for reminiscence, then it would depend on the person that was watching, the places that were important to them. (CS8)* |
|  | Content improvement | *Something different? Yes, they would like something different if it came again, different parts of the aquarium.[…]Yes, a bit of a variety I think is the sense, yes. (CS2)* |
| Potential audiences/suitability | Care home residents, people in need of more exercise, retired community, people with mobility issues, people experiencing pain, hospital patients, rehabilitation, house-bound individuals, those less active. | *I think really it is designed more for nursing homes and places, groups like memory cafes. For a group like us, it’s probably not very suitable to be honest. (CS5)* |
