# Supplementary material for: Developing Digital Platforms to Help Older Adults Stay Physically and Socially Active: Co-Design Study to Develop a Toolkit and Evaluate Its Impact
Source: JMIR Aging. 2026 Aug 27;9:e89785. doi: 10.2196/89785 (PMC13521207; doi:10.2196/89785)
Supplement: Supplementary file 5 [file aging-v9-e89785-s005.docx]

| **Organisation** | **Project Aim** | **Key Impact** |
| --- | --- | --- |
| Weekday Wow | Alexa app for older adults’ health & wellbeing | Reduce loneliness, improve wellbeing, raise awareness on digital inclusion |
| Scottish Football Museum | Reminiscence videos with former players | Emotional connection, therapeutic use in memory groups |
| Sentai | Gamified reminiscence & physical activity tools | Boost fun, intergenerational ties, improve health for limited-mobility users |
| HiCarer | App module connecting older people to local physical activities | Combat loneliness, improve health, integrate into daily care |
| GoodBoost | Digital aqua rehab paddle for aquatic exercise | Enhance strength & balance in pools, reduce fall risks |
| ROVR/Motus | VR tool for social connection in care homes | Improve relationships, reduce isolation, enable long-term VR use |
| danceSing | Enhanced web platform for exercise & singing | Improve usability, grow global user community, provide leading digital resources |
| Mature Movers | Web platform for local & online exercise classes | Increase access, scale nationally, improve older adults’ physical independence |
| Triangular Pixels | VR fitness game tailored to older adults | Promote daily exercise, broaden gaming inclusion, attract investment |
| Age Scotland | Body Boosting Bingo game with improved exercise videos | Include wider ability range, reduce injury risk, enhance inclusivity with subtitles |
