# Supplementary material for: Developing Digital Platforms to Help Older Adults Stay Physically and Socially Active: Co-Design Study to Develop a Toolkit and Evaluate Its Impact
Source: JMIR Aging. 2026 Aug 27;9:e89785. doi: 10.2196/89785 (PMC13521207; doi:10.2196/89785)
Supplement: Supplementary file 6 [file aging-v9-e89785-s006.docx]

**Adaptations**

"So what we're looking into is more classes that are 20 to 30 min. So it's something we're exploring to embed into it, because at the moment we don't have any that last 20 min"

"So it's like I move for 20 min a day. I can do things like gardening and kneeling down. So we've made it a lot more relatable. So we that's how we've done it. So it's similar to exercise difficulty but flipped round."

**Business benefits**

" And I think that's what's really beneficial. You can look at it within 5 min and say, this is what people need.

"This is what people want. Let's go away and build it so. And that's so valuable because people take years iterating ideas. But with this? It doesn't. It takes 5 min to get the necessary information that you need to build. Build an MVP."

"..the stakeholders that I'm sharing with that they really value this research because it shows fraction, and it shows demand "

**Enhanced end-user insight**

"..this is kind of out of our control in a sense, just because some of the deliverers aren't chosen by us. But it's something that we will consider about like creating kind of like a manual of an expectation manual of what people are delivering. So it's not something we'd ever thought about until this. So it's like, okay […] accents are really important element. It's not something we've considered, but how we be, culturally, responsible"

" Yes, organic would have happened, but it would be much longer slower, like prices are just being so much florid. It would have taken a lot more time. I think we probably would have made a few mistakes in the sense of like prioritizing the thing, things that weren't necessary. And then we've just spent that money on that. It wasn't important. So I think that's where the tool gets beneficial."

"I like more stuff, because then I can go back to it. And it's not something I'm gonna look at once. It's something I'm looking at regularly. But for those for people who are just trying to get some data, then maybe they would be like, yeah, okay, we're looking specifically for this"

**Challenges to developmen**t

"..like to realistically create what we've created. It would probably cost us like 30,000 pounds. We've only had 5,000 pounds to play with. So we've had to kind of figure out a way to make what we want to make at this stage with 5,000 pounds and a discount."
