# Supplementary material for: Developing Digital Platforms to Help Older Adults Stay Physically and Socially Active: Co-Design Study to Develop a Toolkit and Evaluate Its Impact
Source: JMIR Aging. 2026 Aug 27;9:e89785. doi: 10.2196/89785 (PMC13521207; doi:10.2196/89785)
Supplement: Supplementary file 7 [file aging-v9-e89785-s007.docx]

**Adaptations**

" And it was the fact that you guys had a had another larger group to be able to feed information which kind of validated some of the stuff that we were getting hints of ourselves."

"The main one from my perspective, from sort of more of a technology focus was really that complexity of the setup. So we've worked quite hard over the last year or so, and from feedback both from you on the care settings up here around, making it as easy as possible."

**Business benefit**

"..removing uncertainty around how to use the technology and providing continual straight forward access to training."

"And what was valuable, really valuable to us, I think in the relationship we had was the what we were seeing, which was in a relatively small number of care settings was amplified and given credibility. Further credibility. You know, if you've only got one result, then you don't know whether that's.."

"..here I think this would also be extraordinarily useful. Would be the people who were right at the very start of the journey. so they haven't even they haven't been to a care setting. They're not, they're thinking of an idea. And they think this might be a a useful way of doing things in any of the ones that you're looking. You know that you've generated for the toolkits. That would be a really good starting point."

"So that just makes it possible to ship it and for other people. So yes, you were right. The anticipation is, it will make it an easier sell, not requiring us to be present."

"..then technology sits on the side even if the tech, even if the Resident loves it, if there isn't a similar level of reward for the care staff. then I think that that technology doesn't get used and I think that the care staff are too often forgotten"

**Enhanced end-user insight**

" And it was the fact that you guys had a had another larger group to be able to feed information which kind of validated some of the stuff that we were getting hints of ourselves."
